# Postdural Puncture Headache after Spinal Anaesthesia in Parturients Undergoing Cesarean Section in the Department of Anesthesia in a Tertiary Care Center: A Descriptive Cross-sectional Study

**DOI:** 10.31729/jnma.8140

**Published:** 2023-05-31

**Authors:** Gajal Lakhe, Pravin Shrestha, Vijaya Duwadi

**Affiliations:** 1 Department of Anesthesia, Manipal College of Medical Sciences, Fulbari, Pokhara, Nepal; 2 Department of Obstetrics and Gynaecology, Manipal College of Medical Sciences, Fulbari, Pokhara, Nepal; 3 Manipal College of Medical Sciences Fulbari, Pokhara, Nepal

**Keywords:** *cesarean section*, *headache*, *prevalence*

## Abstract

**Introduction:**

The post-dura! puncture headache is one of the common complications of spinal anaesthesia. It is one of the most frequent claims for malpractice involving obstetrics anaesthesia. Though self-limiting it is troublesome to the patient. The aim of this study was to find out the prevalence of post-dural puncture headache after spinal anaesthesia in parturients undergoing cesarean section in the Department of Anesthesia in a tertiary care centre.

**Methods:**

A descriptive cross-sectional study was done among parturients who underwent cesarean section under spinal anaesthesia from 27 June 2022 to 19 January 2023 after receiving ethical approval from the Institutional Review Committee (Reference number: MEMG/480/IRC). The pregnant patients aged 18-45 years of the American Society of Anesthesiologists Physical Status II/IIE who underwent elective or emergency cesarean section under spinal anaesthesia were included. A convenience sampling method was used. Point estimate and 95% Confidence Interval were calculated.

**Results:**

Among 385 parturients, the prevalence of post-dural puncture headache was 27 (7.01%) (4.53-9.67, 95% Confidence Interval). A total of 12 (44.44%) cases experienced post-dural puncture headache in the first 24 hours followed by 9 (33.33%) and 6 (22.22%) cases in 48 and 72 hours respectively. Moderate pain was complained of by 3 (11.11%) and 2 (7.41%) cases at 48 and 72 hours post-cesarean section respectively.

**Conclusions:**

The prevalence of post-dural puncture headache after spinal anaesthesia in parturients undergoing cesarean section was similar to studies done in similar settings.

## INTRODUCTION

Spinal anaesthesia (SA) is the most commonly used technique for cesarean section (CS). Post-dural puncture headache (PDPH) is one of the significant complications of SA. The symptoms are caused by the downward movement of the brain and traction on the dura due to low cerebrospinal fluid pressure following leakage of cerebrospinal fluid from the dural hole.^1^ Unfortunately, parturients constitute the highest risk category for PDPH, with the incidence up to 25%.^2^

The female gender, age, and postpartum decrease in intra-abdominal and peridural pressure are risk factors for PDPH in parturients. The postural nature of PDPH prevents parturients from performing routine activities and the inability to look after newborns might precipitate dissatisfaction, anxiety and depression. CS is frequently performed under SA in our institution. Although PDPH is a self-limiting and nonfatal condition, it is troublesome to the patient.

The aim of this study was to find out the prevalence of post-dural puncture headache after SA in parturients undergoing CS in the Department of Anesthesia in a tertiary care centre.

## METHODS

A descriptive cross-sectional study was conducted on a parturient undergoing CS in the Department of Anesthesia of Manipal Teaching Hospital, Pokhara, Nepal from 27 June 2022 to 19 January 2023. Ethical approval was taken from Institutional Review Committee (Reference number: MEMG/480/IRC). The pregnant patients aged 18-45 years of the American Society of Anesthesiologists Physical Status II/IIE who underwent elective or emergency CS under SA were included. Patients having a contraindication to SA, severe cardio-pulmonary disease, patients who developed headache without postural variation, patients who had headache in the pre-operative period and those cases who were converted to general anaesthesia were excluded from the study. Written and informed consent was taken from all patients enrolled in the study. A convenience sampling method was used. The sample size was calculated by using the following formula:


$$\begin{array}{ll} \text{n} & ={\text{Z}}^{2}\times \frac{\text{p}\times \text{q}}{{\text{e}}^{\text{2}}}\\ & =1.96^{2}\times \frac{0.50\times 0.50}{0.05^{2}}\\ & =385 \end{array}$$

Where,

n = minimum required sample size
Z= 1.96 at 95% Confidence interval (CI)
p = prevalence taken as 50% for maximum sample size calculation
q = 1-p
e = margin of error, 5%

The minimum sample size calculated was 385.

A thorough pre-anaesthetic check-up with the relevant investigation was done for all patients. A baseline pulse rate, blood pressure, and oxygen saturation were recorded. An 18 G intravenous cannula was inserted and the ringer's lactate was started before SA. Lumbar puncture was done in sitting or lateral position at L3-L4 or L4-L5 intervertebral space with 25 G Quincke's needle. After assuring a free flow of clear CSF, 1.8 ml of 0.5% bupivacaine heavy with 6 mcg dexmedetomidine was injected. When the sensory blockade of T4 was achieved which was assessed by pinprick sensation, surgery was started. Regular monitoring of pulse, blood pressure and oxygen saturation was done. Any fall in pulse or blood pressure was managed by injection of atropine and injection mephentermine respectively in titrated doses. The standard analgesic regimen was followed in the postoperative period. On the day of surgery, Inj. Ketorolac 30 mg IV tds, Inj. Tramadol 50 mg and ondansetrom 4 mg IV tds, Inj. Meperidine 50 mg and promethazine 25 mg IM sos was prescribed. It was converted to oral medication the next day with tab flexion 1 tab tds and Inj. Diclofenac 75 mg IM sos.

The patients were assessed for headache in the postoperative period at 24, 48 and 72 hours using Visual Analogue Scale (VAS) Scale.^3^ The patients were first educated about the VAS scale, where 0 meant no pain and 10 meant worst, unbearable pain and they were asked to rate their headache on a 10-point scale. Depending on the VAS score, the headache was graded as mild (1-3), moderate (4-7) and severe (7-10). Any associated features such as neck stiffness, tinnitus, hypoacusis, photophobia, and nausea were recorded. The location of headache (localized/generalized) and drugs are given for treatment were also recorded. The direction of the bevel of the spinal needle, a number of attempts to puncture the dura, the approach to dura puncture (midline or paramedian), and the experience of the operator (intern/resident/consultant) were also recorded.

Data were entered and analyzed using IBM SPSS Statistics version 21.0. Point estimate and 95% CI were calculated.

## RESULTS

Among 385 parturients, the prevalence of post-dural puncture headache was 27 (7.01%) (4.53-9.67, 95% CI) cases. A total of 12 (44.44%) cases experienced PDPH in the first 24 hours followed by 9 (33.33%) and 6 (22.22%) cases in 48 and 72 hours respectively.

Mild PDPH was experienced by 12 (44%) cases each at 24 and 48 hours respectively which decreased to 8 (29.62%) cases at 72 hours post CS (Table 1).

**Table 1:** Severity of headache (n = 27).

Severity	24 hour n (%)	48 hour n (%)	72 hour n (%)
Mild pain; VAS (1-3)	12 (44.44)	12 (44.44)	8 (29.63)
Moderate pain; VAS (4-6)	-	3 (11.11)	2 (7.41)
Severe pain; VAS (7-10)	-	-	-

**Figure 1:**
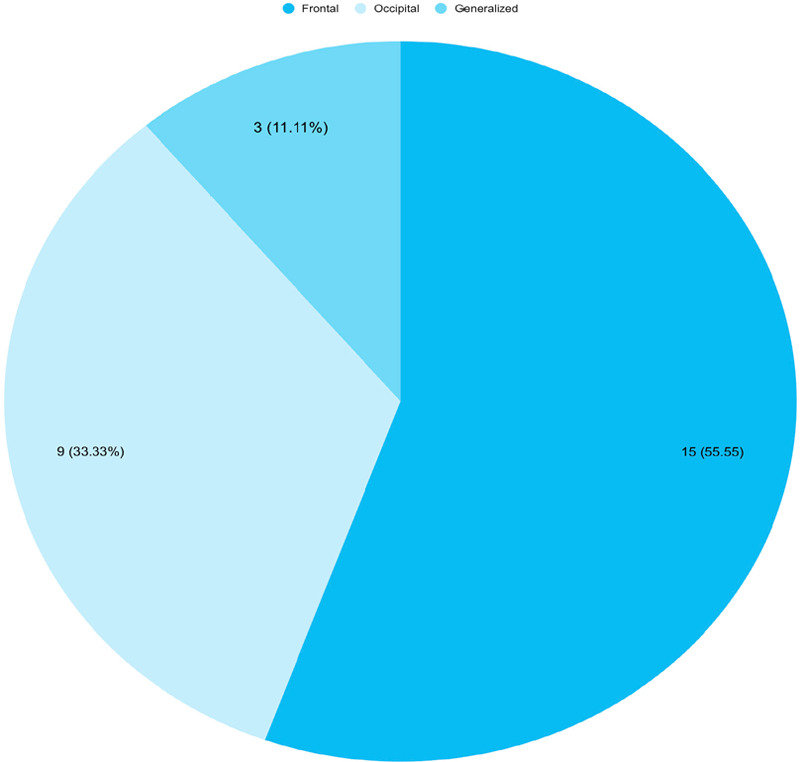
Area of headache (n= 27).

The majority of parturient complained of headache in frontal region followed by occipital region and few complained of generalized headache (Figure 1).

The PDPH was associated with nausea and photophobia in 1 (3.70%) case each at 24 and 48 hours post-CS. The rest of the cases of PDPH were not associated with any other symptoms.

The SA was successfully performed in the first attempt in 22 (81.48%) cases, 3 (11.11%) cases required 2 attempts and 2 (7.41%) cases needed >2 attempts (Table 2).

**Table 2:** Demographic variables of the patients (n= 27).

Variables	Mean±sd
Age (years)	27.63±4.09
BMI^*^ (kg/m^2^)	29.04±3.50
ASA-PS^†^	n (%)
II_E__‡_	11 (40.74)
II	16 (59.26)
**Attempt to dura puncture**	
1	22 (81.5)
2	3 (11.10)
>2	2 (7.40)

* BMI= Body Mass Index

† ASA-PS= American Society of Anesthesiologists-Physical Status

‡ E= emergency.

Out of 27 cases, 24 (88.89%) cases were performed by residents followed by 3 (11.11%) cases performed by faculty. The midline and paramedian approaches to puncture dura were used in 22 (81.48%) and 5 (18.52%) cases respectively. Likewise, the direction of the bevel was upwards in 23 (85.19%) cases and lateral in 4 (14.81%) cases.

## DISCUSSION

The prevalence rate of post-dural puncture headache (PDPH) after CS under SA with a 25 G Quincke needle has been documented in different studies up to 25%.^4^

Our study showed that 27 (7.01%) of the parturient suffered from PDPH in the post-operative period where a 25 G Quincke needle was used. We are in agreement with the findings of several past studies wherein authors have reported the incidence of PDPH with 25 G Quincke needle to be 8.7%, 7.1%, 7.2% and 7.5% in parturient when 25 G Quincke needle was used to perform SA.^5-8^ Our findings are much lower than that reported in another study which was 14.23% with a 26 G Quincke's needle. The probable explanation is that they have used spinal needles of different companies and the stylet protruding beyond the tip of the cutting needle could lead to more dural damage increasing chances of PDPH whereas all of our spinal anaesthesia was conducted with Spinocan (25 G Quincke needle of B-Braun). Regarding the onset of PDPH, our study confirmed that 21 (77.77%) cases suffered from it within 48 hours of SA. A similar study has confirmed that 61.9% of parturients suffered from PDPH within the first 24-48 hours.^9^ Likewise it has been emphasized in one of the past studies that headache characteristically starts 24-48 hours after lumbar puncture and usually lasts one to two days but it may begin as early as one hour after the procedure or may occur beyond one week or sometimes several weeks after it too.^10^

All the patients who developed PDPH had mild to moderately severe headaches, none of the patients developed a severe headache and responded to flexion and paracetamol by oral and/or intravenous routes, oral hydration, and caffeinated drinks. Our findings are similar to the past study conducted in a similar setting using a 25 G Quincke needle.^11^ These patients were managed conservatively with their ongoing pain medication, hydration, and bed rest.

The headache was located in a frontal area in the majority of our cases 55.55%, followed by the occipital region (33.33%) and generalized (11.11%) which is similar to the findings of the previous study where parturient suffered headache predominantly in the frontal (44.44%) region and 11.11% suffered from a generalized headache.^12^ Another study also showed that 57.1% of parturients complained of headache in the frontal region which is similar to our study.^9^

The success of SA in the first, second and more than two attempts in our study was 81.5%, 11.1% and 7.4% respectively which is similar to the past study conducted in a similar setting, in which they reported the first, second and third attempt success rate as 83.5%, 10.2% and 6.1% respectively.^13^ Our institution is a teaching hospital most of the SA has been performed by residents who have spent at least 6 months in anaesthesia which is followed by faculties.

The insertion of the Quincke needle with the bevel parallel to the long axis of the spine most likely results in less tension on the dural hole and subsequently less leakage of cerebrospinal fluid into the epidural space and less PDPH.^9^ In our study, the majority of cases (85.2%) were conducted with bevel perpendicular to longitudinal fibres of the dura. This implies that the prevalence could be further reduced by aligning the bevel parallel to dural fibres in future cases. The use of a paramedian approach to the subarachnoid space has been suggested as a means of reducing PDPH particularly when using cutting needles.^14^ We conducted the majority of SA using a midline approach (81.5%). The para-median approach to dura puncture was used only when there was a failure by the midline approach.

We failed to document the position of the patient while SA was being delivered. There is evidence that dura puncture in the lateral position results in a lesser number of PDPH as cerebrospinal fluid pressure during the lateral position is 20 cm of H_2_O as compared to 40 cm of H_2_O in the sitting position which creates a larger hole in the dura and prolonged leakage of cerebrospinal fluid into the epidural space in the postoperative period.^9^ Likewise, we failed to follow up with the patient beyond 3 days. Hence, further research needs to be conducted in our parturient considering the above factors.

## CONCLUSIONS

The prevalence of post-dural puncture headache after spinal anaesthesia in parturients undergoing CS was similar to studies done in similar settings.

## References

[1] Aftab S, Nur-Ul-Haq S, Ara A, Hassan JA (2009). Post dural puncture headache: comparison of 26G quincke with 25 G whitacre needle for elective caesarian section.. Pakistan Journal of Surgery..

[2] Uluer MS, Sargin M, Akin F, Uluer E, Sahin O (2019). A randomized study to evaluate post-dural puncture headache after cesarean section: comparison with median and paramedian approaches.. Niger J Clin Pract..

[3] Hawker GA, Mian S, Kendzerska T, French M (2011). Measures of adult pain: Visual Analog Scale for Pain (VAS Pain), Numeric Rating Scale for Pain (NRS Pain), McGill Pain Questionnaire (MPQ), Short-Form McGill Pain Questionnaire (SF-MPQ), Chronic Pain Grade Scale (CPGS), Short Form-36 Bodily Pain Scale (SF-36 BPS), and Measure of Intermittent and Constant Osteoarthritis Pain (ICOAP).. Arthritis Care Res (Hoboken)..

[4] Jabbari A, Alijanpour E, Mir M, Bani Hashem N, Rabiea SM, Rupani MA (2013). Post spinal puncture headache, an old problem and new concepts: review of articles about predisposing factors.. Caspain J Intern Med..

[5] Vallejo MC, Mandell GL, Sabo DP, Ramanathan S (2000). Postdural puncture headache: a randomized comparison of five spinal needles in obstetric patients.. Anesth Analg..

[6] Devcic A, Sprung J, Patel S, Kettler R, Maitra-Dcruze A (1993). PDPH in obstetric anesthesia: comparison of 24-gauge Sprotte and 25-gauge Quincke needles and effect of subarachnoid administration of fentanyl.. Reg Anesth..

[7] Batova R, Georgiev S (2019). Impact of spinal needle design and approach to postdural puncture headache and spinal anesthesia failure in obstetrics.. Anaesthesiol Intensive Ther..

[8] Demilew BC, Tesfaw A, Tefera A, Getnet B, Essa K, Aemro A (2021). Incidence and associated factors of postdural puncture headache for parturients who underwent cesarean section with spinal anesthesia at Debre Tabor General Hospital, Ethiopia; 2019.. SAGE Open Med..

[9] Bhusal MK, Rajbanshi L, Amgain G, Amgain K (2017). Incidence and factors associated with post-spinal headache among patient receiving spinal anaesthesia at Bharatpur Hospital Chit wan Nepal.. Eur J Pharm Med Res..

[10] Majd SA, Pourfarzam S, Ghasemi H, Yarmohammadi ME, Davati A, Jaberian M (2011). Evaluation of pre lumbar puncture position on post lumbar puncture headache.. J Res Med Sci..

[11] Biswal D, Mishra J, Sahu BP, Jena S (2023). Postdural puncture headache incidence with 25G and 27G Quincke needles after spinal anesthesia for elective cesarean section.. Asian J Med Sci..

[12] Shah A, Bhatia PK, Tulsiani KL (2002). Post-dural puncture headache in caesarean section-a comparative study using 25G Quincke, 27 G Quincke needle.. Indian J Anaesth..

[13] Thakur S, Sharma A, Kaushal S, Sharma A, Sharma N, Thakur PS (2022). Incidence and risk factors of "postdural puncture headache" in women undergoing cesarean delivery under spinal anesthesia with 26G Quincke spinal needle, experience of medical college in rural settings in India 2019: a prospective cohort study design.. J Pharm Bioallied Sci..

[14] Ghaleb A (2010). Postdural puncture headache.. Anesthesiol Res Pract..

